# Classification of femur fracture in pelvic X-ray images using meta-learned deep neural network

**DOI:** 10.1038/s41598-020-70660-4

**Published:** 2020-08-13

**Authors:** Changhwan Lee, Jongseong Jang, Seunghun Lee, Young Soo Kim, Hang Joon Jo, Yeesuk Kim

**Affiliations:** 1 Department of Biomedical Engineering, Hanyang University, Seoul, Korea; 2 LG Sciencepark, Seoul, Korea; 3 Department of Radiology, College of Medicine, Hanyang University, Seoul, Korea; 4 Institute of Innovative Surgical Technology, Hanyang University, Seoul, Korea; 5 Department of Physiology, College of Medicine, Hanyang University, Seoul, Korea; 6 Department of Orthopedic Surgery, College of Medicine, Hanyang University, Seoul, Korea

**Keywords:** Diagnosis, Computer science, Information technology, Orthopaedics

## Abstract

In the medical field, various studies using artificial intelligence (AI) techniques have been attempted. Numerous attempts have been made to diagnose and classify diseases using image data. However, different forms of fracture exist, and inaccurate results have been confirmed depending on condition at the time of imaging, which is problematic. To overcome this limitation, we present an encoder-decoder structured neural network that utilizes radiology reports as ancillary information at training. This is a type of meta-learning method used to generate sufficiently adequate features for classification. The proposed model learns representation for classification from X-ray images and radiology reports simultaneously. When using a dataset of only 459 cases for algorithm training, the model achieved a favorable performance in a test dataset containing 227 cases (classification accuracy of 86.78% and classification F1 score of 0.867 for fracture or normal classification). This finding demonstrates the potential for deep learning to improve performance and accelerate application of AI in clinical practice.

## Introduction

In general, X-ray images and radiology reports offer complementary information to a physician who wants to make an informed decision. In the classical diagnosis process, the radiologist reads the image and notes the findings, and then the physician makes a corresponding diagnosis and appropriate decision. However, due to the success of deep learning, recent attempts to achieve a high-performance classifier with a deep neural network (DNN) that only inputs images have increased. Since GoogLeNet outperformed humans in 2014^1^, efforts to develop a high-performance classifier in various areas have continued.

The deep learning method is currently popular; however, application to medical fields remains challenging. In particular, protection of patient medical information and unwillingness to share information between hospitals causes difficulty in acquiring a sufficient number of medical images to adequately train DNNs. This leads to performance degradation of DNNs with a relatively large number of parameters, thus requiring a more sophisticated learning algorithm. In addition, the numerous parameters require tuning based on physician assumptions and experience against concrete problems and training datasets, a tedious and resource-intensive task. Meta-learning is a recent technique to overcome (i.e., automate) this problem. The task is also known as “learning to learn” and aims to design models that can learn new tasks rapidly. Several studies have been proposed to apply meta-learning techniques to medical images^2,3^. Kim et al.^2^ used few-shot learning, which is a type of meta-learning method for early diagnosis of glaucoma in fundus images. The authors developed a predictive model based on matching neural network architecture^4^, and showed that the model obtained greater effectiveness than vanilla DNNs. Maicas et al.^3^ presented a simple experiment to demonstrate use of meta-learning for fine-tuning a medical image dataset and demonstrated better classification performance than the current state-of-the-art method.

Recently, the combined modality was shown to be capable of simultaneous use to produce better classifiers than either modality alone^5–7^. In particular, many attempts have been presented to use other image modalities as ancillary information in object detection tasks. In the study by Hoffman et al.^6^, the authors proposed an additional representation learning algorithm that incorporates ancillary information in the form of an additional image modality at training to produce a more informed single-image modality model. Xu et al.^7^ presented pedestrian detection from RGB images with ancillary thermal imaging data. However, these networks require an additional network for hallucination of additional image modality inputs and extensive computation and memory.

A radiology report contains a radiologist’s analysis of findings and is a reflection of the radiologist’s experience and expertise. The report is directly related to the image and serves as a complement to possible missed information from deep learning-based model when using only the image. Therefore, using information that contains both images and radiology reports will improve decision making. To the best of our knowledge, use of radiology reports as additional information in the medical image classification task has not been reported. Thus, we first present an algorithm that uses available paired image-text training data (meta-training set) to learn features from both modalities without an additional hallucination network. When using this approach, a novel deep learning model is produced to operate only over the single-image modality input and outperforms the standard network trained only on image data. Thus, the new method transfers information commonly extracted from text training data to a network that can extract associated information from image counterparts. In a preliminary study, the effectiveness of the proposed meta-learning method at classifying an X-ray image as femur fracture type based on the the Arbeitsgemeinschaft Osteosynthese foundation/Orthapaedic Trauma Association (AO/OTA) classification standard was demonstrated.

## Related works

In several studies, classification of bone fractures based on conventional machine learning pipelines consisting of preprocessing, feature extraction, and classification steps has been addressed. Preprocessing methods include noise reduction, edge detection^8^, and feature extraction methods including Gabor filter to extract textural features of an image^9^. In the classification step, a method using random forest^9^ and support vector machine (SVM) was proposed^10^.

With the advent of deep learning models over recent years, several approaches to classify bone fractures have been proposed. Chung et al.^11^ studied classification of the proximal humerus with the ResNet 152 network^12^, and Lindsey et al.^13^ showed the effectiveness of the 13k wrist X-ray data set for wrist fracture classification. Kazi et al.^14^ attempted to classify proximal femurs based on AO classification standard, which is similar to the model proposed in the present study. These studies have demonstrated the potential of deep learning models, but they require large amounts of data.

In the present study, a meta-learning model that incorporates ancillary information in the form of an additional modality at training time was established. In addition, the model was validated using standard evaluation metrics including accuracy and F1 score, t-distributed stochastic neighbor embedding (t-SNE) to visualize representation vectors, and lesion visualization using gradient-weighted class activation mapping (Grad-CAM).

## Materials and methods

This retrospective study was approved by the Institutional Review Board (IRB) of Hanyang University Medical Center with a waiver of informed consent (HYUH 2019-06-003). All experiments were performed in accordance with relevant guidelines and regulations.

### X-ray images and the radiology reports dataset

**Figure 1:**
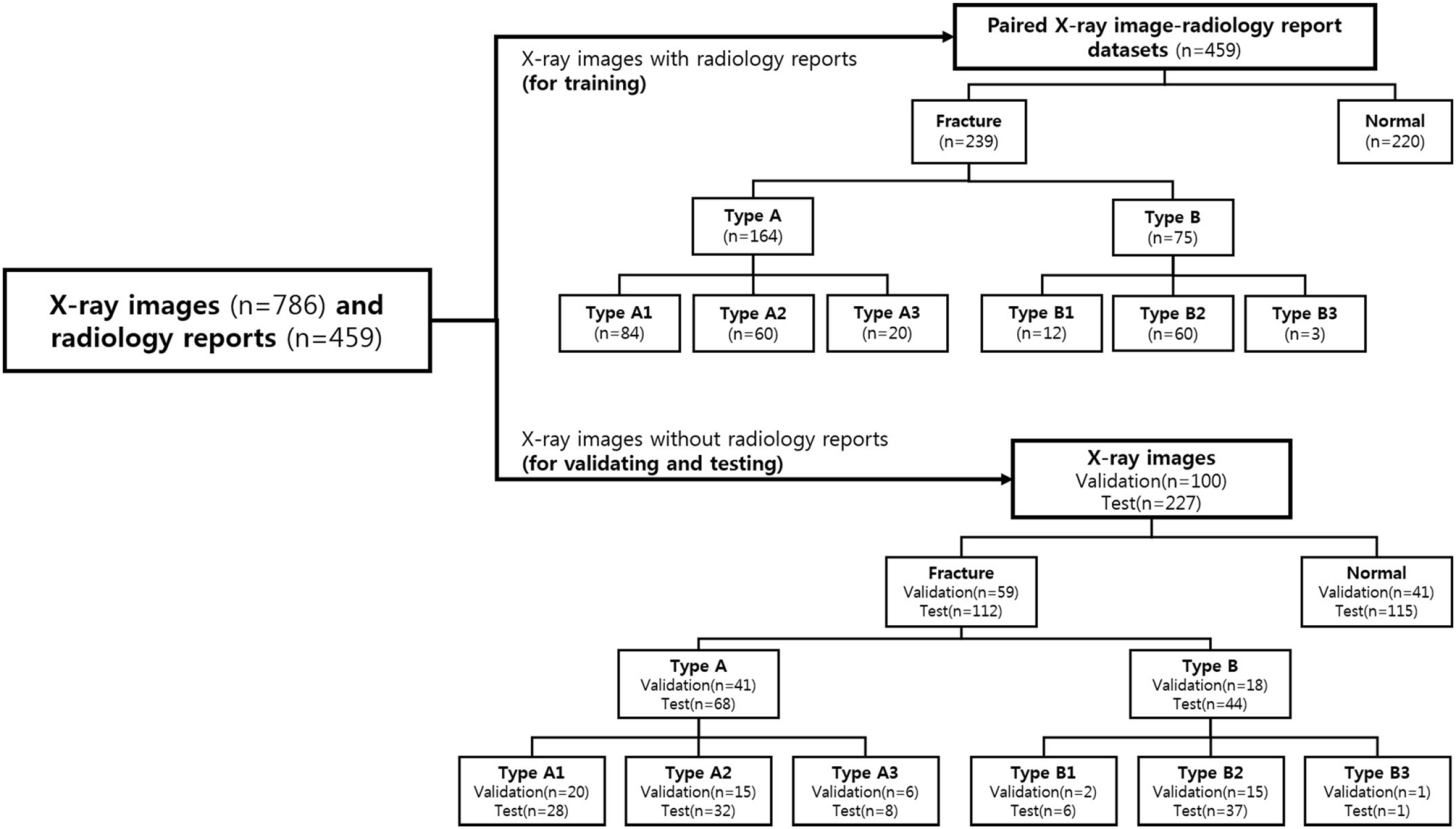
Data characteristics. Number of X-ray images and radiology reports for training, X-ray images for validating, and X-ray images for testing the system.

The institutional review board of our institution approved this study. A total of 786 anterior–posterior pelvic X-ray images and 459 radiology reports acquired from 400 patients was obtained from our institution. From our institutional research database, X-rays were screened to identify cases with or without femur fracture. A digital radiographic examination (CKY Digital Diagnost; Philips, Eindhoven, The Netherlands) included anterior–posterior views of the hip obtained for the patients. The X-ray images and the radiology report from January 1, 2018 through December 31, 2018 were collected separately, and matched by patient ID and study date. The paired data (459 X-ray images and the radiology reports) was used as training sets, and 327 X-ray images which did not match to the reports were used as validation and test sets. The validation and test sets were divided randomly. An experienced surgeon and radiologist reviewed the initially identified cases and confirmed 410 fracture cases and 376 normal cases to be included in the overall dataset. Within the dataset, 59 fracture and 41 normal cases were randomly selected for use as validation data, while 239 fracture and 220 normal cases and the radiology reports were used to train the models. For model testing, 112 fracture cases and 115 normal cases were used (Fig. 1).

The images had varying dynamic range and resolution, and the radiology reports were in a descriptive format. An experienced orthopedic surgeon manually selected the sentences after excluding sentences not written in English and those that contained typos. The radiology reports contain a description of the locations and type of the fractures in the examination. Of a total of 457 overall words from the text dataset, only the 300 most frequent were used. The dataset was annotated into seven classes by an experienced orthopedic surgeon following the AO/OTA classification standard (Supplementary Fig. 1). Several examples of paired X-ray image-radiology report datasets for training are shown in Table 1. In addition, Global Vectors for Word Representation (GloVe) was used to obtain vector representations for words in the radiology reports^15^. GloVe is an unsupervised learning algorithm for generating word vectors by aggregating global word-to-word co-occurrence matrix from a corpus. The resulting vectors show linear substructures of the word vector space. Briefly, the model automatically converts each word in a given sentence to a vector representation. The data distribution between classes was highly unbalanced, with 5 cases for class B3 and 376 for normal class. To balance the training dataset, simple data augmentation was used based on rotation, flipping, and scaling, resulting in four-times images per class, except for the normal class. The augmentation technique was applied only to the training set. To ensure the clinical value of the results, the models were trained on 1,176 augmented datasets and report performances on 227 completely separate test datasets.

**Table 1:**
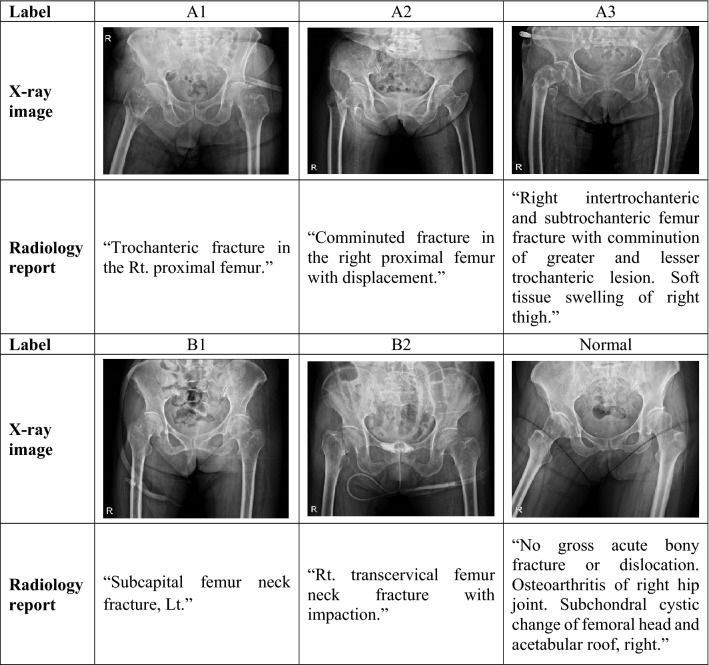
Training examples of X-ray images and the radiology reports.

### AO/OTA classification standard

The AO/OTA classification of fractures is a system for classifying bone fractures by categorizing injuries according to theragnosis of patient anatomical and functional outcome^16,17^ (Supplementary Fig. 1). Fractures of the proximal femur are divided into A type (trochanteric region), B type (neck), and C type (head) based on fracture position. Each type is divided into subcategories (A1, A2, A3, B1, B2, B3, C1, and C2) based on fracture shape. In the present study, C type femur fracture data were not used because they are extremely rare.

### Deep learning model for disease classification in medical images with ancillary information

**Figure 2:**
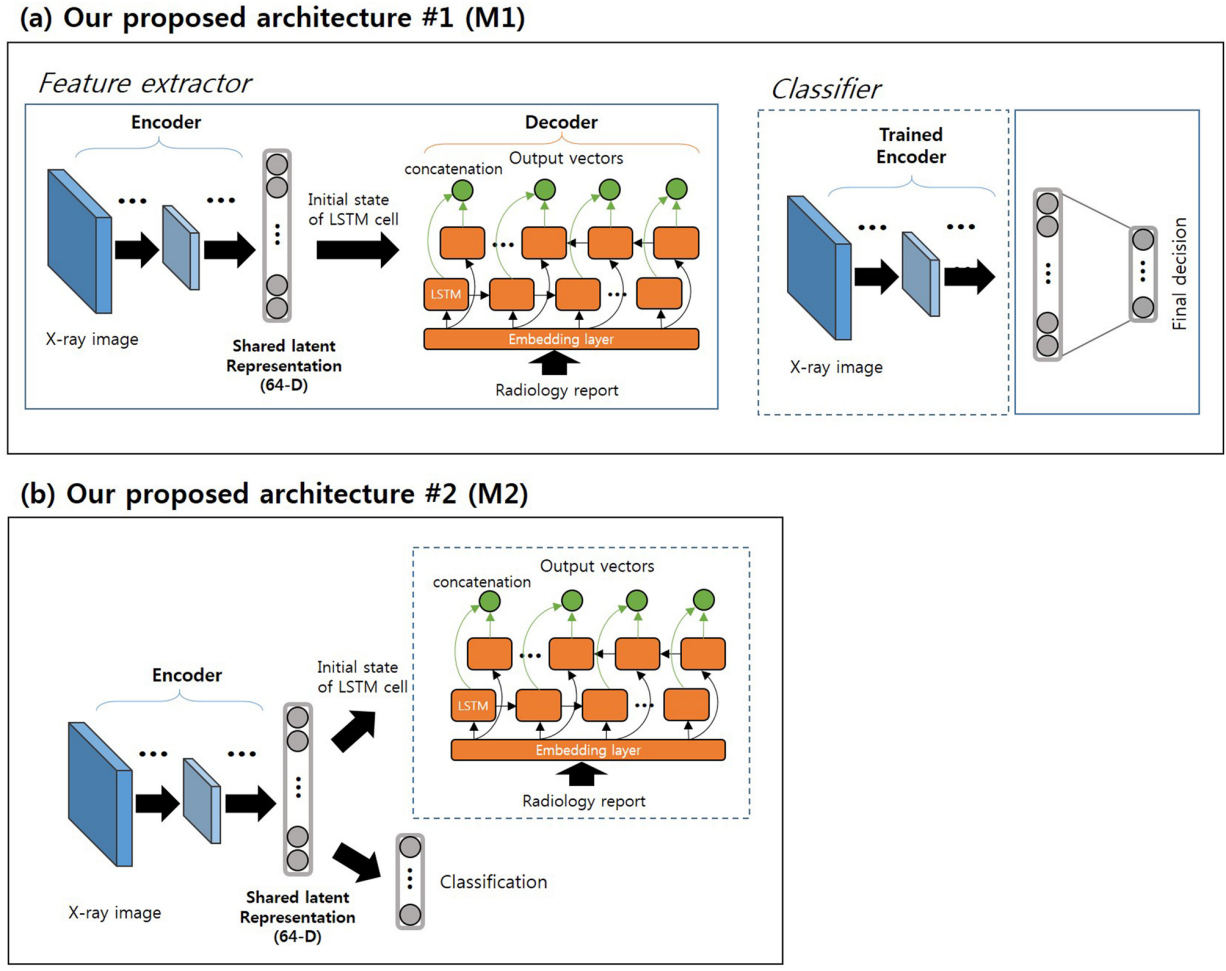
Overview of the proposed models. (**a**) First architecture (M1). The encoder-decoder and the classifier were trained separately. (**b**) Second architecture (M2). The classifier and the decoder were jointly trained from the same latent representation.

**Figure 3:**
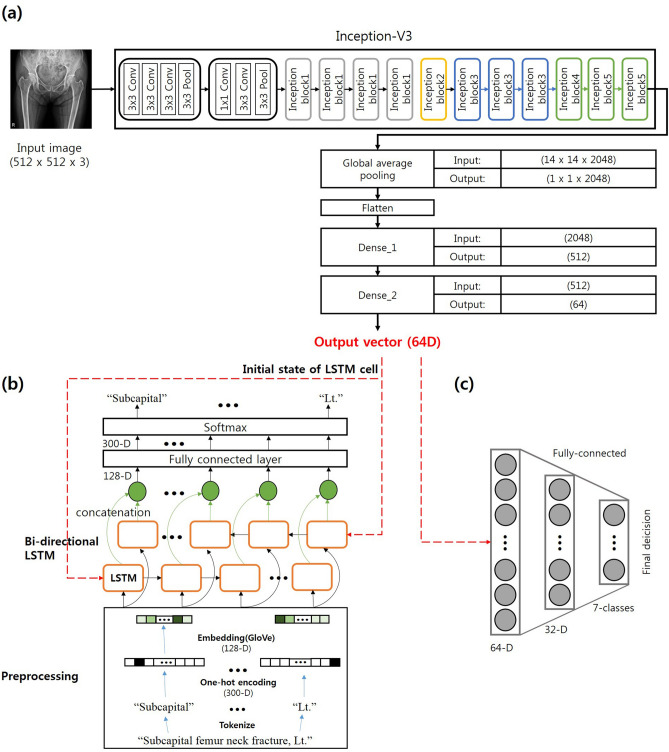
Detail structures of our proposed model. (**a**) Block diagrams of the encoder network topologies with last fully connected layers for Inception-V3^1^. Inception-V3 comprised of a sequence of convolution, pooling layers, and several inception modules. (**b**) Diagrams of the decoder network topologies with preprocessing. (**c**) Classifier model structure, which is comprised of two fully connected layer.

In the present study, two deep learning architectures for use of radiology reports as ancillary information were proposed (Fig. 2). Each has an encoder-decoder architecture and uses the latent representation from both modalities. Our main assumption was that the encoder can compress the input image and use this compressed vector for complete restoration as corresponding text using a decoder to produce a latent representation containing both image and text information. Figure 2a illustrates the first architecture of the proposed model (M1). GoogLeNet (inception v3) was used in the encoder and has been successfully validated for these particular tasks in medical applications^1^. Each input image was resized to 512 × 512 pixels. A bi-directional long/short-term memory (Bi-LSTM) network^18^ was used as the decoder architecture. Each set of text was decomposed by words and then transformed by one-hot encoded vectors to be fed to the input layer of the decoder. The one-hot encoded vectors were converted into 128-dimensional (D) word representation vectors that contained semantic meaning (i.e. “image of a blue car” −  “blue” +  “red” produces vectors close to that produced by “image of a red car”) in the embedding layer and then transferred to the Bi-LSTM cells. The shared representation vector (64-D) from the encoder was used as the initial hidden state of the Bi-LSTM cells, and the number of hidden layers was 40 (i.e., maximum word length of the text in the dataset). After the encoder-decoder structure was trained, an additional classification network was trained using the shared latent representation from the encoder. Empirically, we found that using each dimensions of vector results in the best classification performance. Figure 2b illustrates the second architecture of model (M2). The structure of encoder, decoder, and classification network as in the first architecture was used. The second architecture jointly trained classification task and radiology report restoration task from the same latent representation. At testing, given only an X-ray image, the image was passed through the encoder network and the classifier network to produce scores per category, which were subjected to softmax to produce the final predictions. Specific structures of each module are presented in Fig. 3.

### Network training

In this section, optimization details for the architecture are described. In M1, the encoder-decoder and the classifier were trained separately. A cross-entropy loss between decoder output and the one-hot encoded vector was used; encoder-decoder loss was defined using the following equation:

1 $$ L_{encoder - decoder} (Y^{dec} ,L^{dec} ) = - \sum\limits_{i}^{C} L_{i}^{dec} \log (Y_{i}^{dec} ) $$

where $Y^{dec} ,L^{dec}$ and C are the decoder output vector, the one-hot encoded vector, and the number of hidden units, respectively. Cross-entropy loss for a classifier network was defined as follows:

2 $$ L_{cls} (Y^{cls} ,L^{cls} ) = - \sum\limits_{i}^{C} L_{i}^{cls} \log (Y_{i}^{cls} ) $$

where $Y^{cls} ,L^{cls}$ and C are the classifier output vector, the target vector, and the number of class, respectively. The M2 jointly learns decoder and classifier from latent representation. The loss function was defined as follows:

3 $$ L = L_{cls} + \lambda L_{encoder - decoder} $$

where $L_{cls} ,L_{encoder - decoder}$ and $\lambda$ are the classification loss, the encoder-decoder loss, and weight for encoder-decoder loss, respectively. All models were trained with the Adam optimization algorithm with a learning rate of 0.0001^19^. Dropout^20^, which helps regularize data to reduce overfitting by ignoring several nodes in each layer, was applied to every layer in both the encoder and decoder at a rate of 0.5. We empirically set to 0.5. The training of the architecture included back-propagation and stochastic gradient descent. We also used focal loss^21^ to cope with the imbalance of positive and negative samples. When vanilla cross-entropy is used as the loss function for imbalanced classification, the model is more likely to be affected by a large number of samples, which leads to deviation in the overall learning direction of the model. Details of the loss function is described in Supplementary method.

### Training data augmentation

Data augmentation was performed for the training data by applying geometric transformation (rotation, scaling, and translation) to allow models to learn features invariant to geometric perturbation. Rotation angles ranged from − 10° to 10° with a 1° interval, scaling ratios of heights and widths ranged from 90 to 110% with a 1% interval, and translation parameters ranged from − 10 to 10 pixels in x and y directions with a 1-pixel interval. All parameters were randomly selected in the predefined ranges.

### Experimental environments

The operating system used was Microsoft Windows 7 64-bit professional, and the CPU was an Intel i7-4770k. The main memory size was 32 GB, and an GTX1080TI graphics accelerator with 11 GB RAM was used for parallel processing. The deep learning framework was TensorFlow (version 1.80, Google Brain Team)^22^.

### Gradient-weighted class activation mapping (Grad-CAM)

For the test datasets, the Grad-CAM technique^23^, which generates attention maps highlighting the important regions in images for model prediction to a target label *c*, was performed. The class discriminative localization map Grad-CAM $L_{Grad - CAM}^{c} \in {\mathbb{R}}^{u \times v}$ of width *u* and height *v* for any class *c*, *y*^*c*^ (before the softmax), with respect to feature maps *A*^*k*^ of a convolutional layer, i.e. $\frac{{\partial y^{c} }}{{\partial A_{ij}^{k} }}$, was obtained. The gradients flowing back were global-average-pooled to obtain the neuron importance weights $\alpha_{k}^{c} {:}$

4 $$ \alpha_{k}^{c} = \frac{1}{Z}\sum\limits_{i} \sum\limits_{j} \frac{{\partial y^{c} }}{{\partial A_{ij}^{k} }} $$

This weight $\alpha_{k}^{c} :$ represents a partial linearization of the deep network downstream from *A*, and captures the importance of the feature map *k* for a target class *c*. Next, a weighted combination of forward activation maps was performed, followed by a ReLU to obtain the following:

5 $$ L_{Grad - CAM}^{c} = ReLU(\sum\limits_{k} \alpha_{k}^{c} A^{k} ) $$

Notably, this results in a coarse heat-map of the same size as the convolutional feature map. A ReLU was applied to the linear combination of maps because only the features that have positive influence on the class of interest were relevant i.e. pixels whose intensity should be increased to increase *y*^*c*^.

## Experiments and results

The dataset consisted of 786 X-ray images and 459 radiology reports and was split into training (paired 459 X-ray images and the reports), validation (100 X-ray images), and test (227 X-ray images) sets, and the ratio of fracture to non-fracture cases was similar among datasets. The results were compared using three architectures for the test dataset. The first was GoogLeNet-inception v3 (base network)^1^ used in the encoder of the proposed architectures, and the others were the two proposed architectures (M1 and M2). For the overall classification experiment that was comparable to a prior study^14^, three levels of discrimination were evaluated: (1) fracture versus normal, (2) classification among three groups (normal, A, and B), and (3) classification among seven subgroups (normal, A1, A2, A3, B1, B2, and B3).

**Figure 4:**
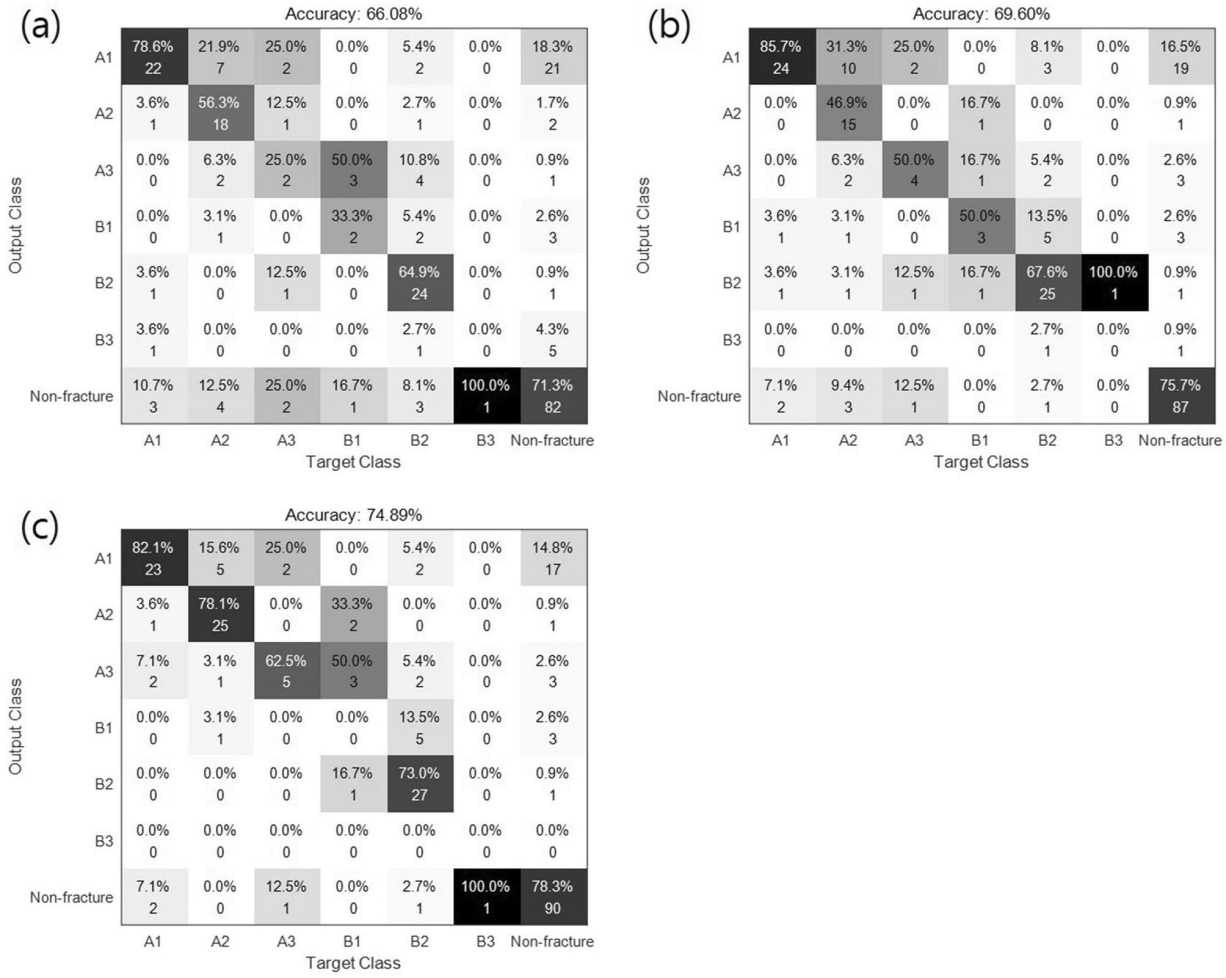
Confusion matrix obtained for 7-class classification in (**a**) GoogLeNet (inception V3), (**b**) proposed model #1 (M1), and (**c**) proposed model #2 (M2).

Figure 4 shows a confusion matrix that represents the actual and predicted classes, where columns are predicted classes and rows are actual classes. The true-positive (TP) measure for each class is the number of positive examples correctly classified using the model, which is each diagonal element of the matrix. The false-positive (FP) measure for each class is the number of classes that are incorrectly classified as positive. The false-negative (FN) for each class is the number of positive classes incorrectly classified as negative, while true-negative (TN) is the number of negative classes correctly classified using the classification model. For evaluation, classification performance was calculated using the following quantitative metrics:

- $$ Accuracy = (TP + TN)/(TP + TN + FP + FN)  $$
- $$ Precision = TP/(TP + FP) $$
- $$Recall = TP/(TP + FN) $$
- $$ F1\;score = 2 \times Precision \times Recall/(Precision + Recall) $$

The performance of the conventional and proposed methods for classification are summarized in Table 2. The base network achieved an overall accuracy of 79.30% on the 2-class discrimination task and an overall accuracy of 66.08% on the 7-class discrimination task. The proposed methods (M1 and M2) showed favorable performance for all the performance metrics. Specifically, M2 showed the highest accuracy (74.89%) for the 7-class task and improved the performance of the conventional method by more than 8.8%. M1 was similar to the conventional method for classification performance in the 7-class discrimination task but showed greater performance for simple tasks (2-class and 3-class). The receiver operating characteristic (ROC) curves for the performance of the three models (base, M1, and M2) for the 7-class discrimination task are shown in Supplementary Fig. 2. Area under the ROC curve (AUC) values obtained for the base model, M1, and M2 were 0.73–0.86, 0.81–0.88, and 0.72–0.90, respectively, after excluding the very rare B3.

**Table 2:** Results for the different models and levels of hierarchical discrimination (2-, 3-, and 7-class).

Model	Measure	2 Class	3 Class	7 Class
Base network (Inception v3)	Overall accuracy	79.30%	73.13%	66.08%
	Avg. F1 score	0.792	0.717	0.458
M1	Overall accuracy	85.02%	79.74%	69.60%
	Avg. F1 score	0.845	0.791	0.493
M2	Overall accuracy	86.78%	82.38%	74.89%
	Avg. F1 score	0.867	0.817	0.501

We also performed five-fold cross validation with paired dataset(459 X-ray images and the radiology reports), not images only. The results are shown in Supplementary Fig. 3 and Supplementary Table 1. The overall performance decrease occurred because the number of training data small, but M1 and M2 still showed better performance than the base model.

Supplementary Fig. 4 is visualization of the latent representation vector embedded in 2D space by the t-SNE for the three models. There was a total of 227 vectors from the test sets, and each class is represented by a different color in the figure. The latent representation vectors from the base network are dispersed, while those from M2 are relatively separate with respect to class label. This indicates that features with greater discrimination are learned using meta-learning methods with radiology reports.

**Figure 5:**
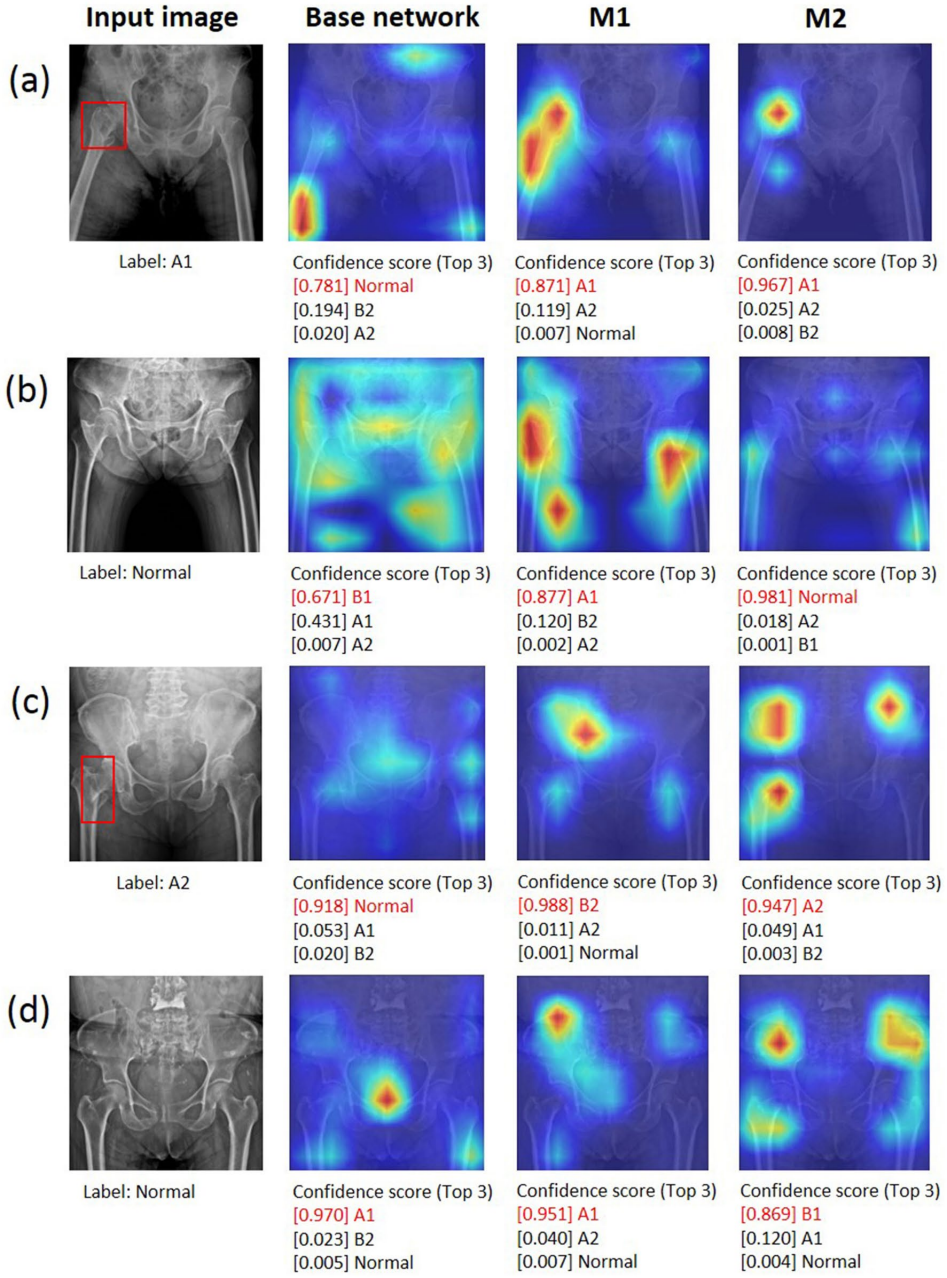
Examples of Grad-CAM-assisted images with the three models. (**a**) For the pelvic radiograph labeled as A1, all models were predicted correctly. (**b**) For the first normal image, only M2 was predicted correctly. (**c**) For the fracture image labeled A2, only M2 was predicted correctly. (**d**) For the second normal image all models were predicted incorrectly. The red boxes are fracture lesions.

While performing inference on a test image, a Grad-CAM^23^ was used to generate a heatmap of hip fracture to provide evidence of fracture site recognition. Figure 5 shows examples of Grad-CAM-assisted images for the three models (base network, M1, and M2). Figure 5a show a case in which all three models produced correct predictions. In Fig. 5b,c, only M2 correctly detected the incorrectly predicted image in the base network and M1. Figure 5d shows a case in which all three models produced incorrect predictions.

## Discussion

In the present study, radiology reports were used as ancillary information for improved classification performance compared to that of X-ray images alone, and deep learning architectures were proposed for incorporating the ancillary information during training. As a preliminary study, the proposed method was effective for classifying an X-ray image as femur fracture type based on the AO/OTA classification standard.

Our work is related to transfer learning and domain adaptation through learning information shared from one task to another. Classical methods consider learning to adapt across distributions through some combination of parameter updates^24^ and transformation learning^25,26^. Christoudias et al.^27^ introduced a method for hallucination of missing modality at training; however, this only applies to weak recognition. Recently, a transformation learning approach has been proposed to use depth information at training for RGB image detection^28^. Similar to our approach, the study learned a single representation from joint modality space.

Our method can also be viewed from the learning with extra or privileged information perspective, which is a type of learning algorithm to generate a stronger model by providing additional information x* about training example ***x***. In this regard, several studies have explored theoretical frameworks^29^, a max-margin framework using bounding boxes and attributes as extra information^30^, and the effects of surface normal during training on detection improvement^31^.

Literature review showed the use of deep learning for classification of femur fractures with the AO/OTA classification standard in only one study. Kazi et al.^14^ presented a method to classify femur fractures on X-ray images using deep learning with an attention module. The method achieved averaged F1-scores of 0.82 in 2-class (fracture and normal) and 0.44 in 7-class (A1, A2, A3, B1, B2, B3, and normal) datasets. Based on the results, the performance of their proposed method was not significantly different from that of the basic model that does not integrate the attention module (only uses Inception V3 network), even in the 7-class task. Conversely, our models showed favorable performance despite using less image data. The results of the present study indicate that the architectures can potentially improve classification performance with high accuracy, F1 score, and AUC value. The first architecture (M1) did not show favorable performance in the complex task (7-class) because the radiology reports contain little information regarding presence and location of fractures. The second architecture (M2) can also be viewed as multi-task learning. In several studies, multi-task learning was helpful to improve generalization performance^32,33^. Although our small dataset is valid, class B3, which is extremely rare compared to other classes, was incorrectly predicted in the three models, indicating that training with very small datasets remains challenging even with ancillary data.

To be more intuitive, we visualize the latent representation vectors from the three models with t-SNE for the training set as shown in Supplementary Fig. 4. In our datasets, B class images have strong edges that are not clearly visible relative to A class images, so that there is a possibility to predict B class images as other classes. In the t-SNE map, the base network does not sufficiently classify the normal class and the B class feature vector region, but the latent representation vectors from the M2 network are sufficiently separated with respect to their class label. We also found that learning with classification task helps to extract grouped latent representation vectors by each class. As can be seen in Supplementary Fig. 4b,c, the representation vectors of the M2 network are more discriminative than the M1 network.

Understanding how a DNN makes predictions is an active research topic in the medical field and may convince doctors the results obtained are valid even though the model may use an incorrect part of the image rather than the true lesion site to produce the answer. Therefore, feature visualization helps in understanding the underlying mechanism of DNNs^34^. In the present study, X-ray images and radiology reports were used for training and X-ray images for testing. Then, Grad-CAM was performed to visualize the class discriminative regions as the fracture sites recognized by the DNN in the images. In Fig. 5a, the three models accurately predicted the fracture class. However, the class discriminative region of the base network did not contain the fracture site, and the confidence score (after softmax) was low. Conversely, in the proposed M1 and M2, the class discriminative regions contained the fracture site and showed relatively higher confidence scores than those of the base network. The images in Fig. 5b do not have femur fractures but have a strong edge due to overlap with abdominal fat. The base network and M1 identified the input images as fracture cases because the class discriminative region was shown with edges from overlap of abdominal fat. However, M2 did not identify that region and predicted the fracture correctly. In Fig. 5c, only the class discriminative region of M2 contains the fracture region. As shown in Fig. 5d, the normal images with strong edges (e.g., overlap of buttocks, pubic tubercles, and sacroiliac joint) distributed in several regions might not be well predicted.

Exploring the relationship between images and natural language has recently attracted significant interest among researchers due to the importance in various applications such as bi-directional image and text retrieval^35,36^, natural language object retrieval^37^, image captioning^38,39^, and visual question answering (VQA)^40,41^. However, using radiology reports as additional information in medical image classification tasks has not been previously reported. We first performed a disease classification task in medical images using the radiology report as ancillary information and showed its effectiveness.

Although the proposed models showed favorable performance, the present study had several limitations. First, the dataset was highly imbalanced. Several methods can be used to reduce this problem, such as oversampling with a data augmentation technique and generating synthetic data^42^. Data augmentation was applied in the present study, and there is a plan to adapt generated synthetic data using a generative adversarial network (GAN) in future research. Second, the generalizability of the system at different institutions was not tested; however, pelvic X-ray images and radiology reports tend to have similar constrained language and content in clinical practice.

In conclusion, we proposed two deep learning architectures for using radiology reports as ancillary information. The model can extrapolate information missing from X-ray images using radiology reports, which is similar to the clinician’s decision making process. Using the general evaluation method for classification and Grad-CAM, the efficiency of the proposed model was demonstrated, and the proof-of-concept solution can be extended to various clinical applications.

## Supplementary information

Supplementary information

## Data Availability

The datasets generated for this study contain protected patient information. Some data may be available for research purposes from the corresponding author upon reasonable request.

## Code availability

The deep learning models were developed using standard libraries and scripts available in TensorFlow. Custom codes for deployment of the system are available for research purposes from the corresponding author upon reasonable request.
