# Supplementary material for: Classification of femur fracture in pelvic X-ray images using meta-learned deep neural network
Source: Sci Rep. 2020 Aug 13;10:13694. doi: 10.1038/s41598-020-70660-4 (PMC7426947; doi:10.1038/s41598-020-70660-4)
Supplement: Supplementary file 1 — Supplementary information [file 41598_2020_70660_MOESM1_ESM.pdf]

Classification of Femur Fracture in Pelvic X-ray Images Using Meta-learned Deep Neural Network

Changhwan Lee[1], Jongseong Jang[2], Seunghun Lee[3], Young Soo Kim[4], Hang Joon Jo[5], Yeesuk Kim[6]

[1] *Department of Biomedical Engineering, Hanyang University, Seoul, Korea*

[2] *LG Sciencepark, Seoul, Korea*

[3] *Department of Radiology, College of Medicine, Hanyang University, Seoul, Korea*

[4] *Institute of Innovative Surgical Technology, Hanyang University, Seoul, Korea*

[5] *Department of Physiology, College of Medicine, Hanyang University, Seoul, Korea*

[6] *Department of Orthopedic Surgery, College of Medicine, Hanyang University, Seoul, Korea*

Address correspondence to: Yeesuk Kim, MD, Ph.D.

Address: Department of Orthopedic Surgery, Hanyang University 222 Wangsimni-ro, Seongdong-gu, Seoul, South Korea, 133-791

E-mail: estone96@gmail.com

Telephone: +82-10-7371-7372

Supplementary Information

Table of Contents

Supplementary Fig. 1: Example proximal femur fracture images with AO/OTA classification standards **3**

Supplementary Fig. 2: ROC curves for (a) base model, (b) M1, and (c) M2 for multilabel classification of the test dataset **4**

Supplementary Fig. 3: Overlapping results of confusion matrix obtained with five-fold cross validation for the dataset of 459 paired X-ray images and the radiology reports. **4**

Supplementary Table. 1: The average accuracy and F1-score obtained with five-fold cross validation for the dataset of 459 paired X-ray images and the radiology reports. **5**

Supplementary Fig. 4: t-SNE visualization of each latent representation vector for (a) Base network, (b) M1, and (c) M2. N=Normal **5**

Supplementary Method: Focal loss for multi-class classification. **6**

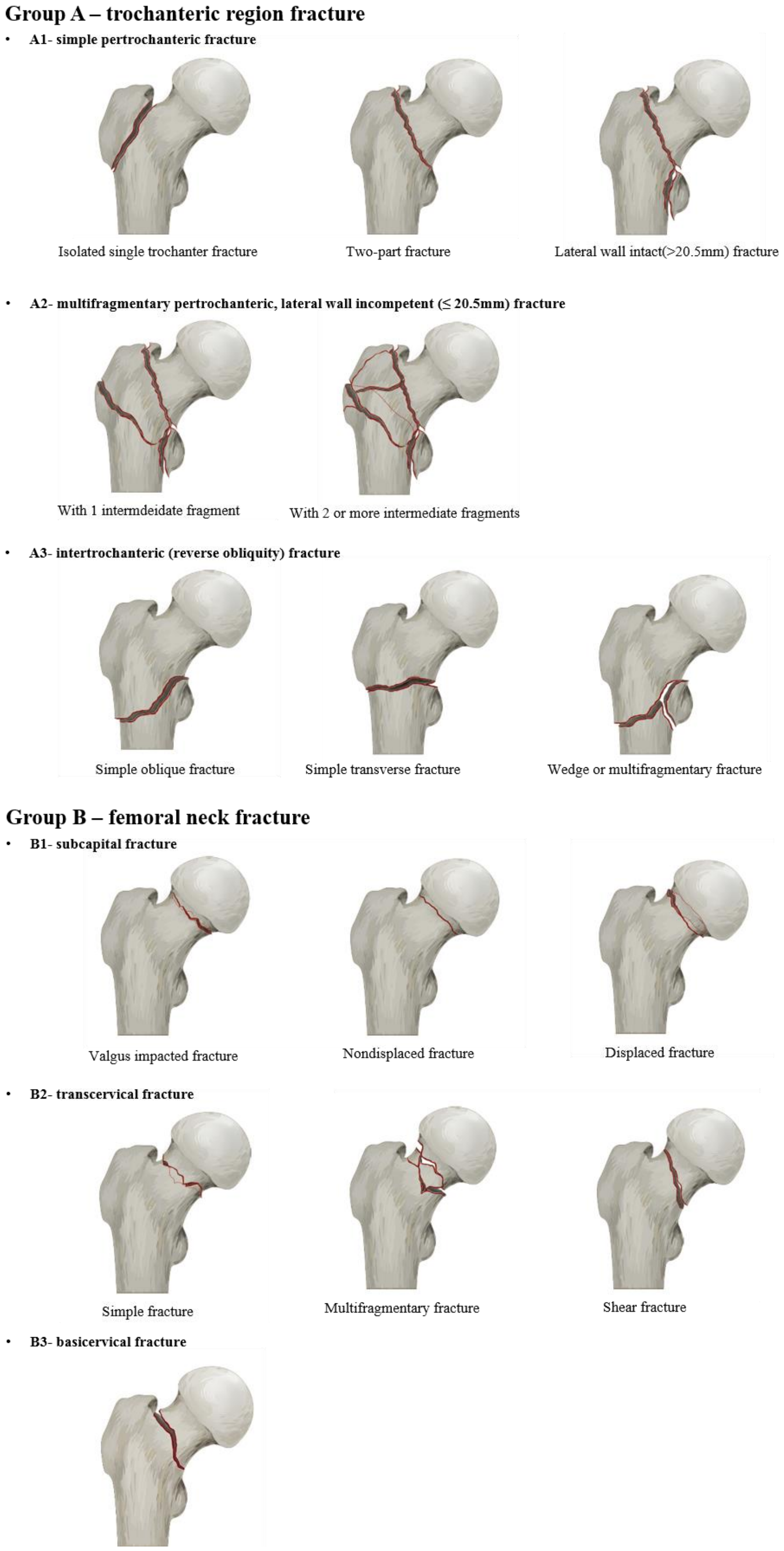

Supplementary Fig. 1 | Example proximal femur fracture images with AO/OTA classification standards. A1: Simple petrochanteric fracture, A2: Multifragmentary petrochanteric fracture, A3: Intertrochanteric (reverse obliquity) fracture, B1: Subcapital femoral neck fracture, B2: Transcervical femoral neck fracture, B3: Basicervical femoral neck fracture.

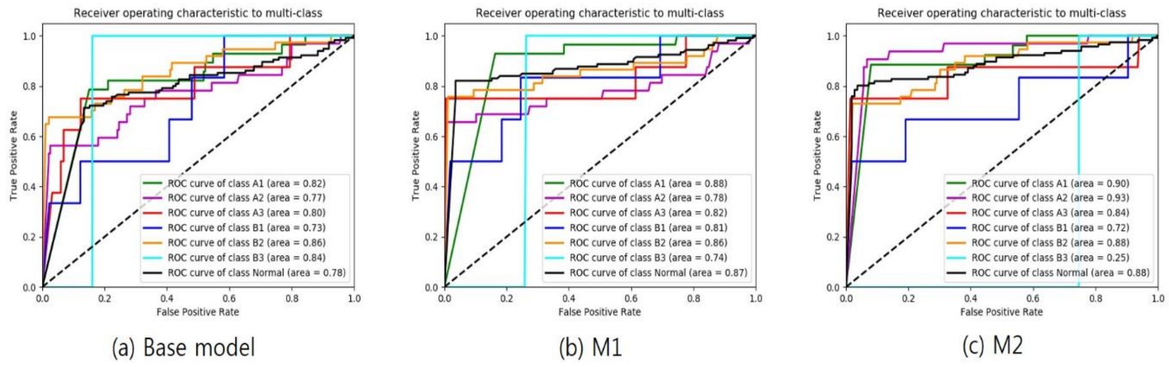

Supplementary Fig 2. | ROC curves for (a) base model, (b) M1, and (c) M2 for multilabel classification of the test dataset.

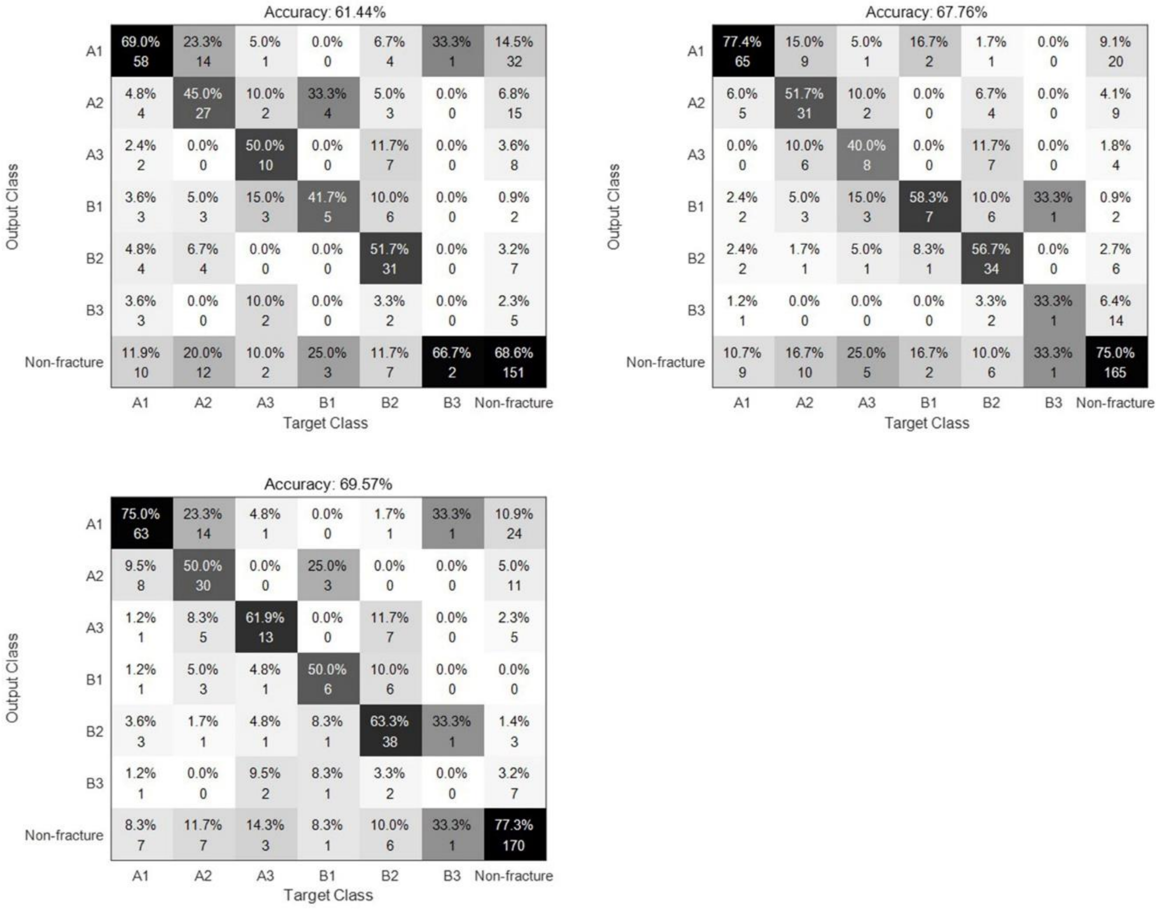

Supplementary Figure. 3 | Overlapping results of confusion matrix obtained with five-fold cross validation for the dataset of 459 paired X-ray images and the radiology reports.

Model	Measure	2 Class	3 Class	7 Class
Base Network (Inception V3)	Avg. Overall Accuracy(%)	76.12 ± 2.04	67.18 ± 1.43	62.24 ± 1.75
	Avg. F1 score	0.768 ± 0.021	0.658 ± 0.016	0.451 ± 0.010
M1	Avg. Overall Accuracy	81.75 ± 2.19	73.85 ± 1.76	67.76 ± 1.68
	Avg. F1 score	0.805 ± 0.022	0.728 ± 0.013	0.487 ± 0.017
M2	Avg. Overall Accuracy	82.58 ± 2.67	76.73 ± 1.40	69.57 ± 1.37
	Avg. F1 score	0.838 ± 0.025	0.767 ± 0.016	0.492 ± 0.014

Supplementary Table. 1 | The average overall accuracy and F1-score obtained with five-fold cross validation for the dataset of 459 paired X-ray images and the radiology reports.

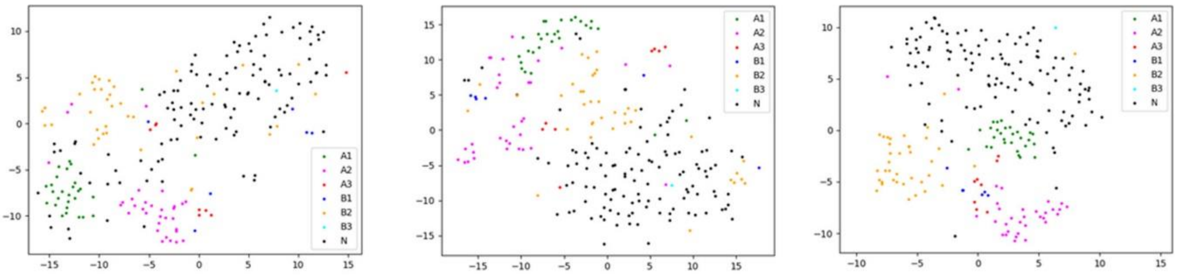

(a) Base network (b) M1 (c) M2

Supplementary Fig. 4 | t-SNE visualization of each latent representation vector for (a) Base network, (b) M1, and (c) M2. N=Normal.

Focal loss for multi-class classification

The original focal loss [21] was designed to address the class imbalance for binary classification, but it can be extended to the multi-class classification tasks. The Cross Entropy (CE) loss for an example is given in equation (1):

$$L_{CE} = -\sum_{i=1}^{C} t_i \log(y_i) \quad (1)$$

where C denotes the number of categories, t_i denotes a real probability distribution, y_i denotes a probability distribution of the prediction. $t_i = 1$ if i belongs to the true label, else it is 0.

The multi-class focal loss can be designed to address the class imbalance via down-weighting easy examples such that their contribution to the total loss is small even if their number is large. In other words, it focuses training on minority examples. A method for addressing class imbalance is to add a modulating factor $(1-y_i)^{\gamma}$ to the cross entropy loss, with adjustable focusing parameter $\gamma \geq 0$. We define the multi-class focal loss, as formulated in equation (2):

$$L_{fl} = -\sum_{i=1}^{C} (1-y_i)^{\gamma}\, t_i \log(y_i) \quad (2)$$

The focusing parameter γ is utilized to control the rate at which easy examples are down-weighted. Especially, when $\gamma = 0$, L_{fl} is equivalent to L_{CE}. The effect of the modulating factor $(1-y_i)^{\gamma}$ is increased with γ. Secondly, when an example is misclassified and y_i is small, the modulating factor nearly tends to 1, and thus the loss is unaffected. As y_i increases to 1, the modulating factor nearly tends to 0 and the loss for well-classified examples is down-weighted. We adopt the multi-class focal loss in our experiments with $\gamma = 1.5$, which is determined empirically.
